# Practical Clinical Lessons on Syphilis and the Genito-Urinary Diseases

**Published:** 1883-10

**Authors:** 


**Practical Clinical Lessons on Syphilis and the Genito-Urinary Diseases.** By FESSENDEN N. OTIS, M. D., Clinical Professor of Genito-Urinary Diseases in the College of Physicians and Surgeons, New York. Surgeon to Charity Hospital, etc., etc. New York: Birmingham & Co. 1883. Six hundred pages, cloth, $4.50.

The work is divided into two parts: Part first treats of syphilis and the genito-urinary diseases. Part second is devoted to the special consideration of gonorrhœa and its sequelæ. The entire work embraces a discussion of every topic in the field of genito-urinary surgery, to which the distinguished author has directed his life. Professor Otis ranks to-day among the most distinguished exponents of urethral surgery, and as the writer found, while in Europe, is as well known, and his writings as highly valued there as in America. His views as to the nature and treatment of gonorrhœa, and urethral stricture, and the normal urethral calibre, have, if not entirely accepted, at least largely modified the previous teaching. The present work is not intended as a full and systematic treatise on syphilis and genito-urinary diseases, but it is rather a clinical and practical presentation of the subject illustrated by reports of cases drawn from the author's rich experience. The book should be read by every physician who treats genito-urinary diseases and syphilis. We believe that they will find in it much of great practical value, and the cases recited, and the principles and practice advocated, will serve to explain many cases otherwise obscure and difficult of management.

